# IDEST: International Database of Emotional Short Texts

**DOI:** 10.1371/journal.pone.0274480

**Published:** 2022-10-07

**Authors:** Johanna K. Kaakinen, Egon Werlen, Yvonne Kammerer, Cengiz Acartürk, Xavier Aparicio, Thierry Baccino, Ugo Ballenghein, Per Bergamin, Núria Castells, Armanda Costa, Isabel Falé, Olga Mégalakaki, Susana Ruiz Fernández

**Affiliations:** 1 Department of Psychology and Speech-Language Pathology, University of Turku, Turku, Finland; 2 INVEST Research Flagship, University of Turku, Turku, Finland; 3 Institute for Research in Open, Distance and eLearning, Swiss Distance University of Applied Sciences, Brig, Switzerland; 4 Leibniz-Institut für Wissensmedien, Tübingen, Germany; 5 Stuttgart Media University, Stuttgart, Germany; 6 Cognitive Science Department, Jagiellonian University, Kraków, Poland; 7 Cognitive Science Department, Middle East Technical University, Ankara, Turkey; 8 Université Paris Est Créteil, Bonneuil, France; 9 Université Paris 8, Saint-Denis, France; 10 Universitat de Barcelona, Barcelona, Spain; 11 Center of Linguistics, School of Arts and Humanities, University of Lisbon, Lisbon, Portugal; 12 Universidade Aberta, Lisbon, Portugal; 13 Université de Picardie Jules Verne, Amiens, France; 14 Sigmund Freud University, Paris, France; 15 FOM University of Applied Sciences, Essen, Germany; Max Planck Institute for Empirical Aesthetics, GERMANY

## Abstract

We introduce a database (IDEST) of 250 short stories rated for valence, arousal, and comprehensibility in two languages. The texts, with a narrative structure telling a story in the first person and controlled for length, were originally written in six different languages (Finnish, French, German, Portuguese, Spanish, and Turkish), and rated for arousal, valence, and comprehensibility in the original language. The stories were translated into English, and the same ratings for the English translations were collected via an internet survey tool (*N* = 573). In addition to the rating data, we also report readability indexes for the original and English texts. The texts have been categorized into different story types based on their emotional arc. The texts score high on comprehensibility and represent a wide range of emotional valence and arousal levels. The comparative analysis of the ratings of the original texts and English translations showed that valence ratings were very similar across languages, whereas correlations between the two pairs of language versions for arousal and comprehensibility were modest. Comprehensibility ratings correlated with only some of the readability indexes. The database is published in osf.io/9tga3, and it is freely available for academic research.

## Introduction

The purpose of the present study was to create a multi-language database of short stories, which convey emotional content that could be classified into different levels of valence and arousal. Reading or listening to text can effectively induce emotions. In experimental studies, stories are often used to induce emotional responses or moods in participants to study how they impact attention and memory [1–4]. Previous research suggests, for example, that while the positive mood is often considered to have a positive impact on cognition, negative affect may also boost attention and subsequent memory [5]. However, even though previous research has established a close link between emotion and cognitive processes [6, 7] very little is still known about the interplay of emotion and cognition during text comprehension [8, 9]. Reading requires coordination of perceptual, attentional, and memory processes, which potentially are influenced by the emotional significance of the stimuli and the emotions experienced by the reader. The International Database of Emotional Short Texts (IDEST) introduced in this paper is a rich database of texts representing various themes and topics and covering a wide range of valence and emotional arousal levels. It aims to allow researchers to examine a variety of research questions related to the role of emotions in text comprehension. As each text is available in two languages (the other being always English), IDEST also serves researchers interested in cross-linguistic comparisons.

In the following, we will first give a brief overview of the previous research on the processing of emotional texts. We will then introduce previously published text databases and finally describe the development of IDEST.

### Processing of emotional texts

Most of the previous experimental studies on emotion effects on reading have studied emotional word recognition [10, 11]. or reading emotional words embedded in simple sentences [12–17]. These studies show that the valence of a word influences reaction times in a lexical decision task [10, 11, 18] and eye fixation times when words are presented in sentence context [12–17]. To conclude, these results indicate that the valence of words plays a crucial role in word recognition, whether presented in isolation or embedded in a sentence context.

Only a few experimental studies have examined how the emotional content of text impacts the processing of text information. In a study combining eye tracking with motion capture recordings to study reading on a hand-held tablet, Ballenghein et al. [19] observed that positive texts were read faster than neutral or negative texts. Moreover, head and tablet motion were reduced when reading emotional (positive or negative) texts compared to neutral texts, indicating that emotional texts induce higher cognitive engagement than neutral texts. In an eye tracking study by Usée et al. [20], participants read short vignettes written based on affective pictures. The results showed that positive vignettes were read faster than negative vignettes. In addition, there was an interaction between valence and arousal on reading speed, indicating that positive emotion increased reading speed only for texts that induce low arousal. Also Child et al. [21] found in a self-paced reading time study that positive texts induced faster reading times.

In summary, these recent studies indicate that positive texts tend to be easier to process than negative or neutral texts. However, the effects seem to depend on various text- and reader-related factors, such as the discursive perspective from which the text is written [21] and the experienced arousal [20]. It is evident that more research is needed to advance the theoretical understanding of how emotions influence the processing and comprehension of text [8, 9]. It is also essential to examine these effects in different languages to address the generalizability of the emotion effects across languages and cultures. The purpose of IDEST is to advance research that examines these (as well as other) issues by providing a set of text materials that cover a wide range of emotional valence and arousal levels, and which are available in more than one language.

### Affective norms for texts

Conducting experimental research requires text materials that are pre-tested for their potential for inducing emotions. While there are affective norms for words [22–34], pictures [35–38] as well as videos and audio (see [39]) to our knowledge there is no such database of text materials representing connected discourse in more than one language.

The existing databases of text materials represent concise texts (individual sentences or short texts) in only one language. The Affective Norms for English Text (ANET; [40]) contain valence, arousal, and dominance ratings for 120 short texts, varying in length from one to a few sentences. The affective norms for Polish short texts (ANPST; [41]) consist of 718 Polish sentences, rated for valence, arousal, dominance, and other emotion-related factors. The Minho Affective Sentences database (MAS; [42]) consists of 192 neutral, positive, and negative declarative European Portuguese sentences, controlled for psycholinguistic characteristics and rated for valence, arousal, and dominance. The Psycholinguistic and Affective Norms of Idioms for German (PANIG; [43]) contains ratings for emotional valence, arousal, familiarity, semantic transparency, figurativeness, and concreteness for 610 German idioms. COMETA is a database of affective and psycholinguistic norms for German conceptual metaphors, containing rating data for 60 metaphorical sentences and their literal counterparts, and 64 stories, half of which contain metaphors [44]. Furthermore, Leveau et al. [24] published emotion ratings for 800 emotional texts in French.

In sum, the existing affective norms for texts mostly represent individual sentences or very short texts and only in a single language. What is needed for research on language and emotions is a database of texts that could be used to study emotion effects during reading or listening to connected narrative texts (see [19–21]). For example, by systematically selecting texts with different valence and arousal levels, it would be possible to examine in more detail how these two dimensions relate to comprehension processes [19, 20]. A text database can also be used to address various other interesting questions. For example, one could manipulate the discursive perspective of the texts (e.g., "I" vs. "You" as in [21]) to study how that impacts experienced emotions and comprehension of texts. Moreover, as the stories in the database cover various themes that are relevant and common to everyday life, one could manipulate the text genre expectations of the reader (fact vs. fiction as in, e.g., [45, 46]) to examine whether that changes how texts are received and comprehended.

It is also important that the texts are available in more than one language to compare these effects cross-linguistically. Simply translating texts into another language for cross-linguistic comparisons without assessing the perceived valence and arousal of the translated text might be problematic, as the text’s potential for inducing emotions might depend on language or vary across cultures. In the present study, we collected sets of short stories written in six different languages (Finnish, French, German, Portuguese, Spanish, and Turkish), which were then also translated into English. Norming data were collected for the original texts as well as for the translations, and we compared the ratings for the original texts and English translations to examine whether ‘something gets lost in translation’. The availability of comparable text materials in two languages allows, for example, cross-linguistic comparisons.

### Overview of the IDEST database

Following the previous text norming studies [40, 41, 43], we approached emotions from the dimensional perspective and measured the affective value of texts on two dimensions: valence (negative–positive) and arousal (calm–arousing) [47]. We chose valence and arousal, as these two dimensions are often used in studies on emotion (see [48]).

Stories often have emotional shifts creating an emotional arc [49] which plays an important role in the emotional reaction of the reader [50]. Reagan and colleagues [49] used computational methods to identify prototypical story plots in a large set of fictional stories, and found six core emotional arcs, which they labelled as Tragedy, Rags-to-riches, Man-in-a-hole, Icarus, Oedipus, and Cinderella. For example, in a tragedy, the story might start off as neutral or even in positive terms, but as the story plot unfolds events spiral down to a negative ending. In a rags-to-riches story, on the other hand, the emotional arc is increasingly positive with a happy ending. Even though it can be expected that the valence and arousal ratings reflect the emotion felt in the end of the story, we also report the emotional arc of each story so that researchers may select stories that best fit their research purposes.

When comparing reading comprehension and processing measures such as reading times of different emotional texts, it is important to control for confounding factors that might impact performance, such as text readability or comprehensibility. Thus, in addition to the ratings of valence and arousal, we also report subjective comprehensibility ratings for all texts included in the database, as well as readability indexes. The Flesch Reading Ease measure is reported for French, German, Portuguese, Spanish and Turkish texts. The measure is not available for Finnish. For the English texts we used the Automatic Readability Tool for English (ARTE; [51]), which is a free tool that calculates a variety of readability formulas for English texts, including the Flesch Reading Ease [52], the Flesch Kincaid Grade Level [53], Automated-Readability-Index [53], the New Dale-Chall Readability Formula [54], the Crowdsourced algorithm of reading comprehension (CAREC; [55]), the Crowdsourced algorithm of reading speed (CARES; [55]), and the Coh-Metrix Second Language Readability Index (CML2RI; [56]).

In order to create the database, we first collected a set of short narrative texts in Finnish, French, German, Portuguese, Spanish, and Turkish. All texts were narratives written from the first person perspective, had varied emotional content, and were approximately 1000 characters long. Texts were collected from various sources to get a rich set of texts covering different topics and themes. To have texts relatable to everyday life, some texts were collected from volunteers who participated in writing competitions. Some texts were written by the researchers to have a more balanced number of negative, neutral, and positive texts. Ratings of valence, arousal, and comprehensibility were then collected from samples of native speakers of these languages. Next, each text was translated into English, and the texts were rated for the same variables by a sample of native English speakers. In the database, we report the ratings for each text in the original language and for its English translation. We also report reliability estimates of the ratings and readability metrics.

In the present paper, we present the valence, arousal and comprehensibility ratings for the English translations and the original texts, and examine the correlations between ratings given to the different language versions (original language vs. English translation) to get a picture of the consistency of emotion ratings across languages. We also examine the relationship between valence and arousal for the original and translated texts, and report the mean valence and arousal ratings for different story types. Finally, we report correlations between comprehensibility ratings and readability indexes.

## Method

### Participants

The rating study of the English translations was approved by the ethics committee for behavioral research at the University of Turku, Finland. For the rating study of the 250 English texts (for details, see below), participants were recruited via www.prolific.co (*n*_*1*_ = 256, *n*_2_ = 250) and via mailing lists at various universities in the UK and the US (*n* = 81). The Prolific participants were recruited in two phases. Participants in the first phase received a reward of £1.03 (£5.00 per hour) and those in the second phase £1.63 (£7.52 per hour). The reward was increased in the second phase to make sure that we reached motivated participants. For our analyses, we only considered participants who provided ratings for all ten texts included in their rating batch, and who reported that they were native speakers of English. This resulted in a final sample of *N* = 573 participants (*M* = 25.36 years, *SD* = 7.45; 64.05% female / 35.60% male / 0.35% other). Of the participants, 61.61% were undergraduate/bachelor students, 20.94% graduate/master students, 9.60% Ph.D. students, and 7.85% reported that they were not students.

### Materials

#### Original texts

The stories were written by adult volunteers or researchers. Authors were instructed to create short (900–1100 characters, except in French 800–1100 characters) emotional narratives written from the first-person perspective. The original texts were edited to correct for spelling and grammatical errors, and in some cases, to match the length and the first person criteria. Next, *valence*, *arousal*, and *comprehensibility* ratings were collected for each text in the original language. We had a joint protocol for collecting the ratings, and each participating team followed this general protocol. First, participants gave informed consent. They then read the texts one at a time in randomized order. After each text, participants rated valence and arousal with the 9-point version of self-assessment manikins [47]. Participants were instructed to "Evaluate how the text made you feel by selecting a picture that best describes your emotion: how positive or negative the emotion induced by the text is and how calm or aroused you feel." Comprehensibility of the text was assessed by asking the participants to "Judge if this text is easy to understand"on a 9-point Likert scale from *1* = *not at all comprehensible* to *9* = *very comprehensible* (except for French, for which the ratings were on a scale from 1 to 7). The details of the data collection had to be adjusted to the context and the resources available in each participating institution, and specific descriptions for how data collection was arranged in each language is provided below.

The 45 *Finnish texts* were written by a student of creative writing based on the text materials originally used in an experimental study by Nummenmaa et al. [57]. The Finnish texts were rated by 77 volunteers recruited from an introductory psychology course. All participants were native speakers of Finnish, five were male, and their mean age was 25.22 years (*SD* = 5.89). Ratings were collected using the Webropol survey tool. Texts were divided into three sets of 15 texts, and each participant rated only one set. Thus, each text was evaluated by 25–26 participants.

The 75 *French texts* were written by adult volunteers (*M*age = 22.28 years, *SD* = 3.21) with an average of 2.58 years (*SD* = 0.56) of university studies. The authors were recruited via social media or personal contact. The French texts were rated by 12 adult volunteers. All participants were native French speakers, seven were male, and their mean age was 22.90 years (*SD* = 1.90). The texts were presented on a computer screen using Microsoft Word, and responses were collected using Microsoft Excel. As the comprehensibility of the French texts was rated on a scale from 1 to 7, the ratings were transformed to a scale from 1 to 9 for the analyses using formula 1 + 8 * (x– 1) / 6, in which x is the original rating.

The 102 *German texts* were collected in writing competitions, one organized in Germany and the other one in Switzerland. In the competition organized in Germany, which was announced via a local web-based participant recruitment system, the authors of the five best stories were rewarded with an online shop voucher. The authors were university students and faculty members (*M*age = 26.10 years, *SD* = 10.65). A total of 55 texts were obtained from the competition. In the Swiss competition, the authors were recruited via social media or personal contact. The authors of the three best stories were rewarded with an online shop voucher. The authors were university faculty and members of the general public (*M*age = 36.82 years, *SD* = 15.78). From the competition organized in Switzerland, we obtained a total of 47 texts. In addition, researchers wrote three texts in order to include more neutral texts. The texts were prescreened to make sure that they were 900–1100 characters long, followed narrative structure, and were written from the first-person perspective. If necessary, they were edited by the researchers to meet these criteria. Then, the texts were rated by 55 university students recruited via a local web-based participant recruitment system. The participants had the chance to win one of 15 10€ Amazon vouchers. Ratings were collected using the survey platform Qualtrics. All participants were native speakers of German, five were male, and their mean age was 23.47 years (*SD* = 2.62). Texts were divided into three sets of texts, and each participant rated only one set. Each text was evaluated by 17–19 participants.

The 36 *Portuguese texts* were written by volunteer university students in the first year of the B.A. in Language Sciences. They were recruited personally in classes at the university (*M*age = 20.80 years, *SD* = 1.65). The texts were prescreened to make sure that they were 900–1100 characters long, followed narrative structure, and were written from the first-person perspective. The Portuguese texts were rated by 63 university students in the third year of the B.A. in Language Sciences. All participants were native speakers of European Portuguese, and six of them were male; their mean age was 22.22 years (*SD* = 1.69). The texts were presented on paper and evaluated with a paper-and-pencil task during a class, at two different times, by two groups of subjects. Each text was evaluated by six to 14 participants.

The 50 *Spanish texts* were written by adult volunteers with a university degree recruited via social media or personal contact (*M*age = 37.89 years, *SD* = 12.64). Of these, researchers wrote two positive, one negative and one neutral text to ensure balanced distribution of text valence. The Spanish texts were rated by 37 university students. All participants were bilingual (Spanish-Catalan), five were male, and their mean age was 25.69 years (*SD* = 5.35). Texts were divided into two sets of texts, and some participants rated only one set, some both sets. Texts were presented on paper, and evaluations were collected with a paper-and-pencil task. Each text was evaluated by 21–26 participants.

The 46 *Turkish texts* were written by adult volunteers (*M*age = 29.30 years, *SD* = 9.30), recruited via social media. The Turkish texts were rated by ten adult volunteers (university students and university staff). All participants were native Turkish speakers, three were male, and their mean age was 38.30 years (*SD* = 11.94). Texts were divided into two sets (23 texts each), and each participant rated a single set online. Thus, each text was evaluated by 5 participants.

#### English texts

The 354 original texts were initially translated into English either by a professional translator (Spanish texts) or members of the research team that were fluent speakers of both languages (the original language and English). The translations aimed to maintain the content and the emotional tone of the text. Problems in translating the text from the original language to English were solved by discussions between the translator and the local research team. Next, we excluded texts based on length; the initial sample of French texts included texts shorter than 900 characters, which was the originally agreed criteria for text length, and 23 French texts were excluded. Three independent raters then read the English translations of the original texts (stories were divided so that two raters read each text) and identified stories that did not follow narrative structure or were incomprehensible in English, which were excluded (in total, 78 texts were excluded on the basis of these criteria). Of the 354 original stories, 250 were retained in the database (see exact numbers of texts retained per language in Table 1). The translations of these texts were proofread (checking for orthographic and grammatical errors) by a professional translator. The English texts are 595–1,371 characters long (*M* = 977.09 characters, *SD* = 146.90 characters). Three independent raters also tagged the main topic of each text; inconsistencies in tagging were resolved by discussion. Finally, the emotional arc of each text was assessed by three independent raters for each text (see Procedure).

**Table 1:** The total number of texts obtained per language and the number and percentage of texts retained in the database.

Language	total # texts	# texts retained	% texts retained
Finnish	45	45	100.00
French	75	46	61.33
German	102	55	53.92
Portuguese	36	30	83.33
Spanish	50	31	62.00
Turkish	46	43	93.48
Total	354	250	70.62

### Procedure

The rating data of the 250 English texts were collected using the Qualtrics survey tool. The texts were divided into 25 sets of 10 texts so that each set consisted of texts that varied in valence and the themes of the stories. The 573 participants were randomly assigned to the different sets. Each of the 25 sets was rated by 20–25 participants. During the rating task, participants first gave their informed consent and then read the texts one at a time, presented in random order. Participants rated *valence* and *arousal* of each text with a 9-point version of self-assessment manikins [47]. *Comprehensibility* of the text was assessed on a 9-point Likert scale from *1 = not at all comprehensible* to *9* = *very comprehensible*. In addition, we asked participants for the following demographic information: education level (undergraduate/bachelor, graduate/master, Ph.D., not student), the field of study, English native speaker (yes/no), age, and gender.

In order to evaluate the emotional arc (i.e., the prototypical story plot) of each text, three independent raters assigned each English text to one of the following categories: 1) Constant / no changes in the emotional tone in the course of the story: the emotional tone can be positive, neutral, or negative, 2) Tragedy: as the story progresses, the story gets more negative and there is a negative ending, 3) Rags-to-riches: as the story progresses, the emotional valence gets more positive and there is a positive ending, 4) Man-in-a-hole: in the course of the story the valence first gets more negative, then there is a change, and the valence gets more positive again with a positive ending, 5) Icarus: the valence first gets more positive, then there is a change, and the valence gets more negative again with a negative ending, 6) Oedipus: the valence first gets more negative, then there is a change, and the valence gets more positive, then there is a second change, and the valence gets more negative again with a negative ending, 7) Cinderella: the valence first gets more positive, then there is a change, and the valence gets more negative, then there is a second change, and the valence gets more positive again with a positive ending, and 8) no clear story arc. All other categories except for the “constant / no changes” and “no clear story arc” categories were based on the story arcs established by Reagan et al. [49]. The two new categories were added to cover all possible text types. To help with the task, raters were given pictures of story arcs representing each of the categories. Two raters rated all 250 texts, and six raters rated 30–46 texts each so that each text was categorized by three independent raters. In the database, the stories are assigned to the story arc category voted by two out of the three raters. When all three raters assigned a text to a different category, the story was categorized as "no clear story arc". None of the texts was initially categorized as "no clear story arc" by the raters.

We calculated the Flesch Reading Ease for French, German, Spanish, Portuguese, and Turkish texts (the formula has not been adjusted to Finnish). The Flesch Reading Ease for French texts was calculated with the formula: 206.835–1.015 * (total number of words / total number of sentences) - 84.6 * (total number of syllables / total number of words). Note that the number of syllables does not take into account elisions. For German [58], Spanish [59, 60] and Portuguese [61] the index was calculated with web-based software tools. Note that the tool for Portuguese was developed for Brazilian Portuguese. For Turkish, Flesch Reading Ease was calculated using a formula by Ateşman [62].

The readability indexes for the English texts were computed with the Automatic Readability Tool for English (ARTE; [51]). It reports the following readability measures: the Flesch Reading Ease [52], the Flesch Kincaid Grade Level [53], Automated-Readability-Index [53], the New Dale-Chall Readability Formula [54], the Crowdsourced algorithm of reading comprehension (CAREC; [55]), the Crowdsourced algorithm of reading speed (CARES; [55]), and the Coh-Metrix Second Language Readability Index (CML2RI; [56]).

### Statistical analyses

For the analyses, we used the R statistical software (Version 3.6.2; [63]). The descriptive statistics and intraclass correlations were calculated with the R package *psych* (Version 1.9.12.31; [64]), for correlations we used the package *Hmisc* (Version 4.3.0; [65]), for multilevel analyses we used the *lme4* package (Version 1.1–27.1; [66]), for the estimation of the ICC—corresponding to an R^2^—we used the package *performance* (Version 0.9.0; [67]), and the density figures were created with the package *tidyverse* (Version 1.3.0; [68]). The scatterplots representing the association between valence and arousal were created with the R package *yarrr* (Version 0.1.5; [69]).

We report two types of intraclass correlations (ICCs). First, we used ICC as an index of interrater reliability [70]. ICC is a descriptive statistic that describes how strongly the ratings of objects, in our case the texts, resemble each other [71]. In other words, it describes the consistency of ratings given by multiple raters for multiple texts. We computed the ICC for each rater group based on a two-way random effects model and mean ratings (i.e., ICC (2, *k*); [70]) and calculated a mean across the rater groups for each variable. To calculate the means, the ICC was transformed with Fisher’s z. The calculation of the ICC permits negative values. Therefore, we calculated the average ICC by setting all negative values to zero [72] to get reliability estimates that vary between 0 and 1.

Second, we calculated the ICC for each measure (valence, arousal, comprehensibility) based on a linear mixed effect model with random intercepts for texts, raters, and language. The model estimates the proportion of variance explained by each of the grouping factors, and the resulting ICC can be interpreted as an *R*-squared value. In the text, we will refer to this ICC value obtained from the multilevel model as ML-ICC to distinguish it from the reliability estimate.

## Results

The IDEST database is available at osf.io/9tga3, and it contains 250 texts in the original six languages, and the means and standard deviations of valence, arousal, and comprehensibility ratings for each original text. Also, Flesch Reading Ease indexes are reported, except for the Finnish texts. The database also includes the English translations, and the means and standard deviations of the valence, arousal, and comprehensibility ratings and readability indexes for the translations. We also included tags for the topics addressed and the emotional arc category for each text.

In the following, we first describe the texts included in the IDEST by reporting the descriptive statistics for valence, arousal, and comprehensibility ratings for the original texts and the English translations. In order to examine how well the ratings translate from one language to another, we also examine the correlations of the ratings between language versions. We then report correlations between valence and arousal ratings, and examine their associations to emotional arcs. Finally, we examine the correlations between readability indexes and comprehensibility ratings.

### Valence

The descriptive statistics for the valence ratings for the English translations and the original texts are presented in Table 2. The average valence rating of all English texts was 4.62 (*SD* = 1.67). The story with the most negative valence rating of 1.24 (*SD* = 0.89) was about sexual abuse. The story with the most positive valence rating of 7.88 (*SD* = 0.99) described an unexpected salary rise. Examples of positive, neutral and negative texts are given in the S1 Appendix. The average ICC(2,k) for the valence ratings of the English texts was .97, and .83-.99 for the original texts (see Table 3), indicating high reliability.

**Table 2:** Means, standard deviations, and minimum and maximum values of valence for all texts, separately for the English translations and the original language versions.

Language	Number	English translations				Original texts			
of origin	of texts	*M*	*SD*	*Min*	*Max*	*M*	*SD*	*Min*	*Max*
All	250	4.61	1.67	1.24	7.88	4.68	1.98	1.17	8.61
Finnish	45	5.15	1.80	2.17	7.88	5.19	2.10	1.77	8.00
French	46	4.79	1.78	1.96	7.35	4.66	1.90	1.25	7.42
German	55	4.23	1.63	1.24	7.48	4.23	2.10	1.17	8.61
Portuguese	30	4.94	1.77	2.29	7.32	5.23	2.04	2.00	8.50
Spanish	31	4.62	1.81	1.90	7.45	4.66	2.19	1.41	8.15
Turkish	43	4.13	1.00	2.44	6.54	4.39	1.42	2.00	7.20

**Table 3:** Reliability of valence, arousal, and comprehensibility ratings for English texts and the texts in their original language as averages of ICC(2,k).

			Valence			Arousal			Compreh.		
Language	Groups	Raters / group	M	Lb	Ub	M	Lb	Ub	M	Lb	Ub
English	25	20–25	.97	.93	.99	.87	.71	.96	.80	.55	.94
Finnish	3	25–26	.99	.97	.99	.96	.93	.99	.54	.22	.82
French	1	12	.96	.94	.97	.87	.81	.92	.81	.71	.88
German	3	17–20	.98	.97	.99	.92	.85	.96	.83	.68	.92
Portuguese	6	6–14	.95	.84	.99	.48	.03	.94	.53	.11	.94
Spanish	3	21–27	.96	.87	.99	.70	.37	.96	.75	.49	.96
Turkish	2	5	.83	.68	.92	.67	.37	.85	.44	.13	.74

*Note*. Groups = number of rater groups, Lbound = Lower bound, Ubound = Upper bound, Compreh. = Comprehensibility.

The mean valence of the stories in the original languages (*M* = 4.68, *SD* = 1.98) was very close to the ratings of the English translations, and the correlation between the valence ratings was high, *r* = .92 (see Table 4). Except for the Turkish texts (*r* = .64), all correlations between the valence ratings of the English texts and the valence ratings in the original language were between *r* = .91 and *r* = .97. ML-ICC values of the valence ratings indicated that texts account for most of the variability (.59), whereas raters (.03) and the language of the text (.00) account for a very small proportion or none of the variance.

**Table 4:** Correlations between ratings given to English translations and original texts, and the ML-ICC values for valence, arousal, and comprehensibility.

	Valence				Arousal				Compreh.			
		ML-ICC				ML-ICC				ML-ICC		
Language	*R*	T	R	L	*r*	T	R	L	*r*	T	R	L
All	.92*	.59	.03	.00	.40*	.16	.22	.13	.53*	.12	.30	.07
Finnish	.97*	.65	.03	.00	.89*	.32	.23	.08	-.05	.00	.61	.17
French	.91*	.59	.02	.00	.46*	.13	.27	.14	.34*	.06	.37	.01
German	.96*	.61	.02	.00	.59*	.19	.20	.07	.69*	.12	.34	.01
Portuguese	.94*	.57	.02	.01	-.07	.06	.29	.00	.36	.05	.28	.22
Spanish	.97*	.66	.01	.00	.40*	.08	.25	.14	.73*	.10	.45	.13
Turkish	.64*	.28	.09	.00	.26	.13	.23	.02	.32*	.09	.24	.10

Note. *p < .05. ML-ICC = intraclass correlation in a multilevel model (R^2^), *r* = Pearson correlation, T = texts, R = raters, L = language, Compreh. = Comprehensibility.

**Fig 1:**
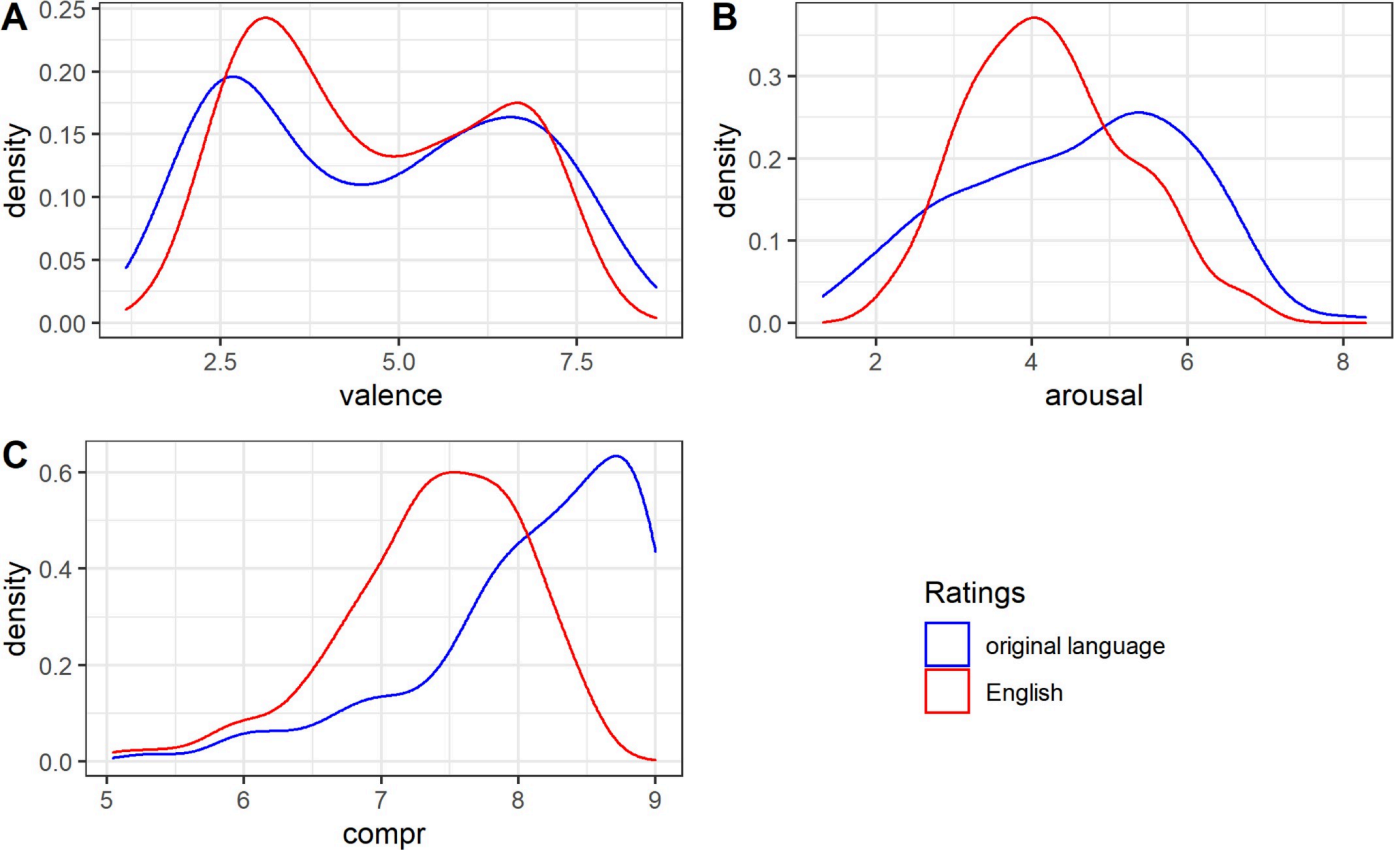
Distributions of the valence, arousal and comprehensibility ratings for original texts and English translations. (A) Valence ratings. (B) Arousal ratings. (C) Comprehensibility ratings.

The distributions of the valence ratings of the English translations and the texts in the original language were very similar (see Fig 1), both showing a bimodal density distribution. This indicates that the texts represented more positive or negative texts than neutral texts.

### Arousal

The descriptive statistics for the arousal ratings for the English translations and the original texts are presented in Table 5. The average arousal rating of the English texts was 4.22 (*SD* = 1.03). The story with the lowest arousal rating of 1.95 (*SD* = 1.75) was about cooking, and the story with the highest arousal rating of 6.88 (*SD* = 1.48) described getting bit by a snake. Examples of low and high arousing texts are given in the S1 Appendix. The ICC(2,k) for the arousal ratings of the English texts was .87, and .48-.96 for the original texts (see Table 3).

**Table 5:** Means, standard deviations, and minimum and maximum arousal ratings for all texts, separately for the English translations and the original language versions.

Language	Number	English translations				Original texts			
of origin	of texts	*M*	*SD*	*Min*	*Max*	*M*	*SD*	*Min*	*Max*
All	250	4.22	1.03	1.95	6.88	4.57	1.44	1.31	8.28
Finnish	45	4.54	1.50	1.95	6.88	3.54	1.44	1.31	6.31
French	46	4.09	0.94	2.46	6.83	5.41	1.12	2.92	7.92
German	55	4.22	1.00	2.17	6.19	5.11	1.35	1.58	8.28
Portuguese	30	4.06	0.70	2.68	5.50	4.30	1.03	2.63	6.83
Spanish	31	4.12	0.71	2.95	5.30	5.38	0.92	3.93	7.15
Turkish	43	4.21	0.90	2.87	6.23	3.64	1.20	1.80	5.80

The mean arousal for the texts in the original language was very close to the English translations (*M* = 4.57, *SD* = 1.44). The correlation between the arousal ratings of the English texts and the texts in their original language was *r* = .40 (see Table 4). Portuguese and Turkish texts showed weaker correlations (*r*’s < .27) than the other texts. The ML-ICC values of the arousal ratings (see Table 4) showed that on average, raters accounted for a bigger proportion of the variance (.22) than texts (.16), indicating that there was significant variability in how different raters evaluated the arousal of the texts. Also, language of the text explained some (.13) of the variance in arousal ratings.

The distributions of the arousal ratings of the English translations and the texts in the original language were both near to normal distributions (see Fig 1).

### Comprehensibility

The average comprehensibility rating of the English texts was 7.39 (*SD* = 0.67) (see Table 6). The story with the lowest comprehensibility rating (*M* = 5.04, *SD* = 2.20) describes somebody’s study career at a university, and the story with the highest comprehensibility rating (*M* = 8.54, *SD* = 0.78) describes a person receiving a wage raise. Examples are given in the S1 Appendix. The ICC(2,k) of the comprehensibility ratings of the English texts was .80, and .55 - .94 for the original texts (see Table 3).

**Table 6:** Means, standard deviations, and minimum and maximum values of comprehensibility ratings for all texts, separately for the English translations and the original language versions.

Language	Number	English translations				Original texts			
of origin	of texts	*M*	*SD*	*Min*	*Max*	*M*	*SD*	*Min*	*Max*
All	250	7.39	0.67	5.04	8.54	8.10	0.78	5.22	9.00
Finnish	45	7.99	0.34	7.17	8.54	8.78	0.18	8.40	8.96
French	46	7.31	0.47	6.35	8.18	7.74	0.72	5.22	8.78
German	55	7.40	0.65	5.10	8.52	7.64	0.76	5.83	8.63
Portuguese	30	7.25	0.49	6.43	8.29	8.61	0.46	7.50	9.00
Spanish	31	7.57	0.54	6.22	8.41	8.41	0.57	5.96	8.88
Turkish	43	6.78	0.75	5.04	7.95	7.76	0.81	5.40	8.80

The mean comprehensibility (*M* = 8.10, *SD* = 0.78) of the stories in the original languages was comparable to the English translations, and the correlation between the comprehensibility ratings of the English texts and the texts in their original language was *r* = .53 (see Table 4). The Finnish and Portuguese texts differed from the other languages in that correlations were low (*r* = -.05 and .36) between the ratings for the original texts and English translations; this is likely due to a ceiling effect and restricted variance in the comprehensibility ratings. Raters accounted for a significant proportion of variance in the comprehensibility ratings (.30), while texts (.12) and language (.07) had smaller contributions to the variance.

The distributions of the comprehensibility ratings of the English texts (Fig 1) showed a normal distribution. However, the comprehensibility ratings of the texts in their original language demonstrated a bimodal right-skewed distribution, indicating that quite many texts were rated as highly comprehensible in the original language.

### Relationship between valence, arousal, and story arc

**Fig 2:**
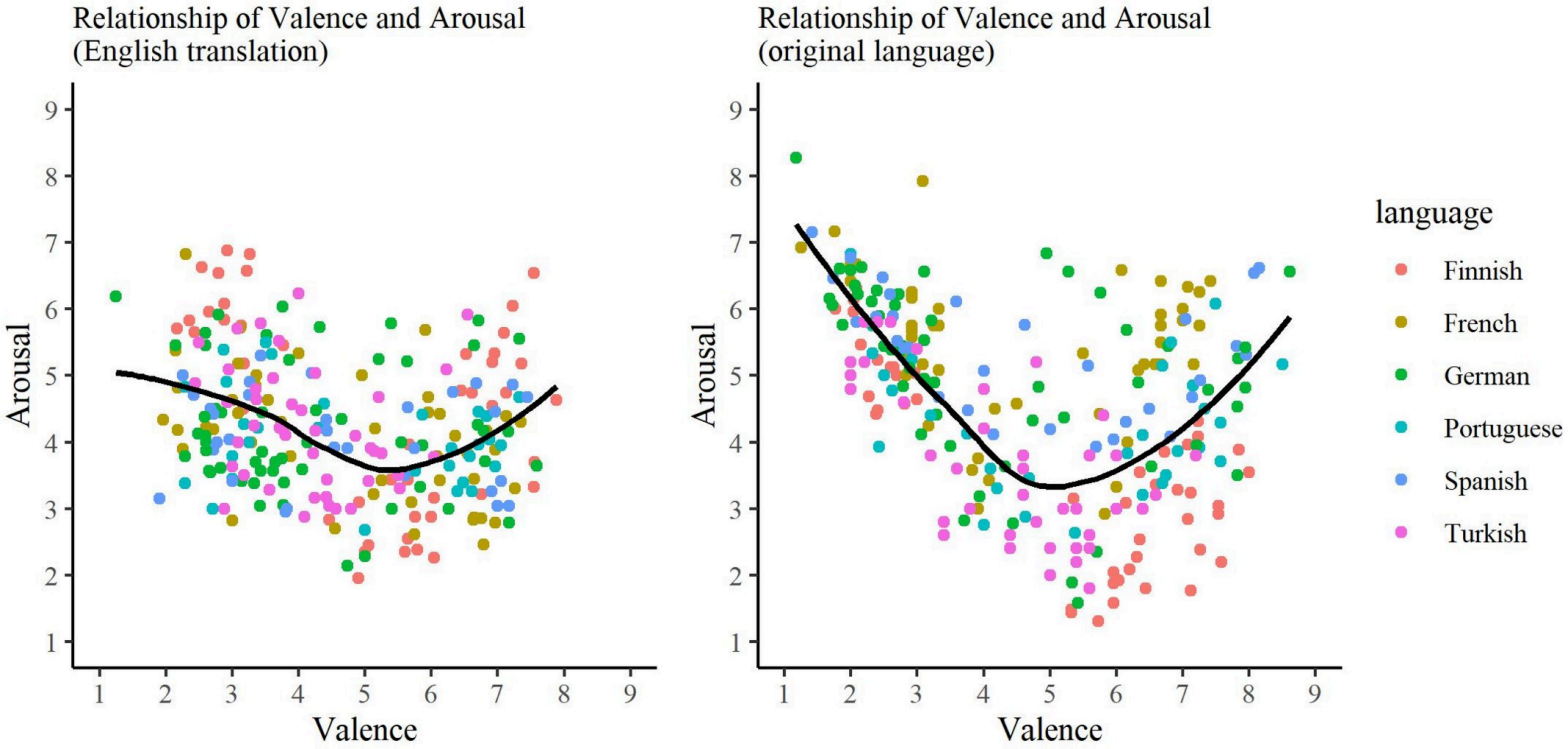
A scatterplot of the relationship between valence and arousal ratings for the English translations (left panel) and original texts (right panel). The different colors correspond to the six languages of the original texts.

The relationships between valence and arousal ratings separately for the English translations and the original texts are presented in Fig 2. The relationship between valence and arousal shows a U-shape for both the English and the original language texts, indicating that positive and negative texts tend to be more arousing than neutral texts. However, the U-shape is flatter for English translations than for the original texts.

The mean valence and arousal ratings for English texts representing different story arcs are presented in Table 7. Overall, it seemed that the valence ratings corresponded with the typical ending of each story type. Tragedies were rated as negative, and Rags-to-riches stories as positive. As for the more complex story plots, Man-in-a-hole stories were rated as overall more positive than Icarus stories, although there was some overlap in the valence ratings of these categories. The few Oedipus and Cinderella stories differed in valence to the expected direction, negative-ending Oedipus texts being rated as negative and happy-ending Cinderella stories as positive. Finally, the stories that had a constant emotional tone or did not have a clear emotional arc represented a wide range of valence values.

**Table 7:** Means, standard deviations, minimum and maximum values of valence and arousal ratings of texts as a function of story type.

		Valence				Arousal			
Story type	N	*M*	*SD*	*Min*	*Max*	*M*	*SD*	*Min*	*Max*
Constant	54	4.88	1.47	2.29	7.48	3.45	0.89	1.95	5.83
Tragedy	69	2.95	0.59	1.24	4.56	4.73	1.03	3.00	6.88
Rags-to-riches	50	6.72	0.59	5.14	7.88	4.28	0.87	2.85	6.54
Man-in-a-hole	19	5.17	1.11	3.18	6.96	4.73	0.83	3.42	6.23
Icarus	18	3.67	0.88	2.18	5.13	4.22	1.04	2.70	6.54
Oedipus	2	3.34	0.36	3.08	3.59	5.51	0.28	5.32	5.71
Cinderella	5	5.76	1.29	4.25	7.05	3.78	0.89	2.61	5.04
No clear emotional arc	33	4.58	1.23	3.00	7.23	4.01	0.77	2.82	5.61

In arousal ratings, the differences between story types were not that clear. The negative Oedipus stories were more arousing than the happy-ending Cinderella stories, and constant stories included more low arousal stories than other story types, probably due to the neutral texts included in this category.

### Readability indexes

Table 8 shows the means of eight different readability scores for the English texts. The values of the readability indexes show that most texts were easy or very easy to read, and, on average, most of the texts should be understandable for an average student in Grades 3 to 7. Five of the seven readability scores correlated, although only modestly (*r*’s > .13 - .21), with the rated comprehensibility of the texts.

**Table 8:** Means, standard deviations, and minimum and maximum values of the readability scores for the 250 English texts and their correlations (*r*) to the comprehensibility rating.

	*M*	*SD*	*Min*	*Max*	*R*
Flesch Reading Ease	81.07	8.18	50.38	98.13	-.13*
Flesch Kincaid Grade Level	5.47	1.99	1.00	15.42	.17*
Automated Readability Index	4.77	2.42	-0.26	16.71	.17*
New Dale Chall Readability Formula	6.16	0.87	1.20	8.48	-.01
CAREC	0.10	0.06	-0.16	0.23	-.10
CARES	0.46	0.10	0.23	0.75	-.20*
CML2RI	24.96	5.37	11.63	38.22	-.21*

*Note*. * p < .05.

The mean Flesch Reading Ease values for the texts in the original languages are presented in Table 9. The correlations between readability scores and comprehensibility ratings vary widely across languages, *r*’s = -.41 - .30. Across all texts in the database, the Flesch Reading Ease score does not correlate with the mean comprehensibility rating (*r* = .03).

**Table 9:** Means, standard deviations, and minimum and maximum values of the Flesch Reading Ease scores for the original texts and their correlations to the comprehensibility rating.

Language of origin	*M*	*SD*	*Min*	*Max*	*R*
All	65.54	13.81	29.18	93.58	.03
Finnish	-	-	-	-	-
French	47.05	10.25	29.18	73.72	-.13
German	73.57	8.30	57.51	93.58	-.41*
Portuguese	70.37	7.75	55.20	85.20	.05
Spanish	68.56	8.90	47.39	84.22	-.18
Turkish	69.51	11.34	33.35	89.67	.30*

Note. * *p* < .05.

## Discussion

In the present study, we report a database of 250 emotional stories (IDEST) that contain two versions of each text: the originally written version in one of the six languages (Finnish, French, German, Portuguese, Spanish, and Turkish) and their counterparts in English. The texts vary in both valence and arousal and are mostly rated high in comprehensibility. As most of the texts were collected from writing competitions in which the instruction was to “write an emotional story”, it is understandable that markedly emotional (either positive or negative) texts are overrepresented. Notably, the database also contains emotionally neutral texts, allowing researchers to compare emotionally valenced and neutral texts in future research.

In the following, we will first discuss the correlations of the valence, arousal, and comprehensibility ratings across languages. We will then consider the limitations of the study and present some recommendations for future research.

### Ratings of valence, arousal, and comprehensibility across languages

The comparisons of the ratings given for the original and the translated texts indicate that the valence of a text was rated very similarly across each pair of languages. Most of the variance in the valence ratings was related to differences between texts, whereas raters and languages contributed very little to the total variance. This result aligns with previous research comparing the results of an automated sentiment analysis conducted for social media posts written in Arabic and their English translations [73]. This study showed that the sentiment analysis conducted on the English translations quite accurately predicted the emotion ratings given by human raters of the original Arabic texts. In other words, valence information was retained in translated texts, as in our study.

For arousal ratings, the consistency across languages was not quite as good as for valence. A significant portion of the variance in arousal ratings was explained by differences between raters, which was also indicated by variance in the reliability of the arousal ratings. This naturally impacts the observed correlations. There are several different reasons that may contribute to the low consistency in arousal ratings. First, as pointed out by a reviewer, reading a story does not elicit as strong an arousal response as, for example, riding a ghost train. Evaluation of arousal requires sensitivity to one’s bodily reactions, and assessing arousal is difficult if those signals are weak. Second, variability in arousal ratings is understandable considering the varied topics of the texts–some stories might induce high arousal in some readers but not in others. For example, a story that yielded relatively high variance in arousal ratings depicted a situation in which the protagonist is about to give a public speech but at the last minute notices that they have lost their notes. People who have performance anxiety might experience this as a highly arousing story. The same story might not be as emotion-provoking for others, even though everybody probably agrees that this is a negatively valenced story. Third, individual differences in mood or other contextual factors might impact the intensity of the arousal induced by text. Future studies could examine in more detail how individual differences in mood or sensitivity to emotions impact evaluations of text emotionality.

The observed U-shaped relationship between valence and arousal replicates findings reported in previous studies [33–34, 44, 74] and indicates that highly emotional texts (either positive or negative) tend to be more arousing than neutral texts (see also [75]). The current database contains enough texts representing different arousal and valence values, allowing, for example, to design experiments in which valence and arousal of the texts are orthogonally manipulated. Interestingly, the U-shaped curve was flatter for the English translations than it was for the original texts. This finding probably stems from the relatively modest consistency of the arousal ratings across languages versions. In other words, while the valence information seems to transfer across languages, ratings of arousal might be language- or culture-specific and show more individual variability, as discussed above.

As for the comprehensibility ratings, it should be noted that overall, the comprehensibility of the English texts was high, with a minimum rating of 5 on a scale from 1 to 9. The restricted variance might impact the correlation of the comprehensibility ratings across languages, as well as the correlation between comprehensibility and readability indexes. Moreover, intraclass correlations showed that a significant portion of variance in comprehensibility ratings was related to differences between raters, and there was quite a lot of variance in the observed reliability of the comprehensibility ratings. This indicates that there was individual variability in how comprehensibility of the texts was scored, possibly reflecting that some raters might have understood ‘comprehensibility’ as referring to the textual and stylistic features of the texts, and others to the content or topics of the texts. There also was some language-related variance in the comprehensibility ratings, suggesting that as the texts were originally written in different languages and may contain culture-specific content, some translated texts might describe events that are not readily comprehensible, even though the texts were pre-screened for comprehensibility in English.

The high readability indexes, based on purely textual features and not semantics, indicate that the English texts should be easy to read. The low correlations between comprehensibility ratings and readability indexes might thus reflect that the readability indexes mainly reflect the lexical and syntactic features of the texts, while comprehensibility may capture semantically related aspects. In other words, a text might represent an unfamiliar situation written in simple language, resulting in a low comprehensibility score but high readability score. The correlation between Flesch Reading Ease index and comprehensibility for the original language texts varied across languages, and across all texts the correlation was close to zero. However, one should be cautious in comparing the readability indexes across languages, as they do not necessarily take into account the specific characteristics of the languages, as for example, elisions when calculating the number of syllables, or typical word length. This explains why the Flesch Reading Ease scores were overall lower in the original language texts than in the English translations.

### Limitations of the study and recommendations for future research

One limitation of the IDEST database is that there was methodological variation in how the ratings for the original texts and the English translations were collected. Due to the differences in available resources, ratings for the original texts were collected in various ways (paper-and-pencil tasks or different digital platforms), and the number of raters varied between languages. These methodological differences should be considered when comparing the ratings of original texts and English translations. For example, the low correlations between original texts and English translations in arousal ratings for Turkish and Portuguese could be due to the low number of raters per text in these languages. If there are individual differences in how readers experience arousal in response to different texts, as we speculate above, the reliability of the arousal estimate in these languages is then necessarily low.

Another point to consider is that we chose to measure the emotional valence and arousal of the texts and not the specific emotion categories that the texts might represent. While texts can induce basic emotions like anger, sadness, or happiness [76, 77], a text may also induce more complex emotions arising from the reader’s evaluation or appraisal of the reading experience (e.g., fascination or suspense, see [78]). It has been suggested that emotion categories are context-dependent, and that it might be hard to find universal categories that generalize across situations [79, 80]. As our aim was to create a database of texts representing different languages, we chose the dimensional approach [48] instead of trying to define emotion category labels representing the vast array of complex emotions that the texts could induce. Future studies could examine in more detail the emotion categories that the texts fall under and the degree to which they are generalizable across languages.

The database contains texts covering a wide range of valence levels, from very negative to very positive texts. Some texts have a clear emotional arc (e.g. an emotional shift from neutral or even positive, respectively, to negative), and our results show that the valence ratings reflect the emotion induced by text at the end of the story. A dramatic shift might be an important aspect in creating a strong emotional reaction in the reader [50]. How the emotional state of the reader evolves is an interesting question but hard to measure, as one would need a setup in which changes in experienced emotions are tracked during the course of reading. We hope that our database helps in conducting research like this in the future—there certainly is a great need for both methodological developments on how to measure transitions between emotional states during the course of reading and theoretical work on how the changing or evolving emotions impact text comprehension and related processes.

Another factor likely to contribute to the ratings of the texts included in the database is that the texts are written from the first person perspective (‘I’). We considered that this would be the most natural perspective for the authors when writing about emotional experiences. Research by Brünye and colleagues [81], however, suggests that the personal perspective (‘You’) might be more efficient than the first person perspective (‘I’) in inducing emotional engagement and ‘simulation’ of the emotional state of the protagonist in the readers. Thus, the valence and arousal ratings in our database probably reflect the lower bound of experienced emotion induced by text. One potential future use of our database is in studies systematically examining the impact of discursive perspective: one could manipulate the perspective to examine how it influences the experienced emotion as well as the processing and memory for text [21].

Finally, it should be noted that the texts included in the database represent various topics or themes (e.g., death, romance, events with friends and family), which may be fictional or non-fictional. We did not collect information about whether the stories describe events that really happened or not. As genre expectations have been shown to impact memory representations constructed for text [45, 46], one potentially interesting use of the database would be to manipulate reader expectations and examine whether that influences the experienced emotion and the processing of text.

## Conclusions

The IDEST database contains short emotional texts originating from different sources covering different topics, normed for valence, arousal, and comprehensibility. Thus, it provides researchers a rich database, which can be used to examine different research questions. The database is open for research use and published at osf.io/9tga3, and we hope that it will be of use to researchers who are interested in the intricate interplay of emotions and language processing. The multilingual nature of the database allows cross-linguistic comparisons and benefits international collaborative research by providing comparable text materials in more than one language.

## Supporting information

S1 Appendix (DOCX)
