# Correction: IDEST: International Database of Emotional Short Texts

**DOI:** 10.1371/journal.pone.0315614

**Published:** 2024-12-06

**Authors:** The PLOS ONE Staff

The Data Availability statement for this paper is incorrect. The publisher apologizes for the error. The correct statement is: All relevant data for this study are publicly available from the OSF repository (https://osf.io/9tga3/).
