# Supplementary material for: IDEST: International Database of Emotional Short Texts
Source: PLoS One. 2022 Oct 7;17(10):e0274480. doi: 10.1371/journal.pone.0274480 (PMC9544016; doi:10.1371/journal.pone.0274480)
Supplement: S1 Appendix — (DOCX) [file pone.0274480.s001.docx]

**Appendix**

**Example of a negative text (mean valence rating 2.14):**

I try to sit as far as possible from my bus neighbor: he smells terribly bad ... Not a simple, pungent smell, not an ordinary cheese smell; no, a throaty smell, mix of rotten fish and dried vomit ... Hm, precisely, I observe it and this supposition seems to me more and more plausible. His greenish complexion and his foul breath suggest that he is not at his best. I do not seem to be the only one disgusted by this terrible smell. On the other side, a little girl is holding her nose. The bus takes a turn. My neighbor takes a deep breath, but it's too late. Stirred by a contraction of his abdomen, he vomits with force. His head is thrown back. He puts his hand in front of his mouth, but that is not enough to stop this yellow liquid. The lumps of his food slip between his fingers to find themselves on my knees. It's my turn to be shaken by spasms and rush to the exit.

**Example of a neutral text (mean valence rating 5.00):**

I’m in a grocery store and push the shopping cart along the aisle. I have arrived just before rush hour, so there’s no crowd or much noise. I only hear typical grocery-store noises and ads blaring from the loud speakers. I glance at my shopping list, which I wrote that morning. I already bought the ingredients for dinner earlier, so all I need on this trip is some items for the fridge. From the fruit section, I pick up some bananas and a few apples. I weigh the fruit and put it in the shopping cart. From the cold shelves, I pick up a couple of milk cartons and some yogurt. From the next shelf, I get some butter and a piece of cheese. I continue to the bread shelves, then stop to decide whether I should get my usual loaf or try some novelty bread from the bakery. I end up trying the novelty bread and put it in my shopping cart. “I should pick up some drinks for the weekend,” I think to myself as I continue.

**Example of a positive text (mean valence rating 7.88):**

I sit in an open office, immersed in my work. It’s a tranquil afternoon at my workplace – phones ringing every now and then, fingers tapping on keyboards, and the coffee maker gurgling. My boss walks into the corridor and stops to say “Hi” to me. I stop working, and we chat a little. My boss mentions a previous project, which I finalized. I’m proud of my work. Suddenly, my boss smiles and offers his hand: “We’ve been very happy with your work.” I shake his hand, slightly puzzled. My cheeks get red, and my heart is pounding. The boss continues: “The board has decided that now would be a good time for a raise – we can talk about that in my office later today.” I can’t hold back a wide smile. I tell him how much I like my job and how happy I am to be appreciated. My boss smiles, nods, and continues walking. I look around, and one of my colleagues gives me a thumbs-up, while another shouts out his congratulations. I grin happily in response, then return to work.

**Example of a low-arousal text (mean arousal rating 1.95):**

I open the fridge and look for something to eat. I feel like eating something light, but tasty. There is a carton of milk in the door and a packet of margarine on the upper shelf. In the vegetable box, there are some tomatoes and a cucumber I just bought. I take the milk, margarine and vegetables to the kitchen counter and close the fridge. I reach up on my tiptoes to get the granola box and bread from the cupboard, then place them on the counter as well. I cut a few thick slices of bread and put them in the toaster, then put the rest of the loaf in a bag. While the bread is in the toaster, I cut some tomato and cucumber slices. I get a bowl from the cupboard and pour granola and milk into it. When the bread is toasted, I put the bread on a plate and spread margarine and the sliced vegetables on them. I carry the granola and sandwich to the table and sit down to enjoy my meal.

**Example of a high-arousal text (mean arousal rating 6.88):**

I’m camping with my friends on a summer weekend. After hiking for an entire day, we stop for the night and set up camp beside a beautiful lake. We put up our tent in a small clearing near the woods and crawl into our sleeping bags, exhausted. It is midnight when I suddenly wake up in the darkness. It takes a while to remember where I am. I feel some movement next to my thigh and feel a shiver up my spine. I try to stay still, even though my heart is pounding, and drops of sweat appear on my forehead. I carefully look into my sleeping bag. Something moves inside it, and I blink my eyes to get used to the darkness. Slowly, my sight gets sharper, and I can see a long, shiny snake resting on my thigh. It’s hissing and licking its tongue. I gasp for air, and I can no longer hold back my panic. I scream and try to get out of the sleeping bag. I feel like I’m stuck because I zipped the sleeping bag all the way to the top.

**Example of a medium-comprehensibility text (mean comprehensibility rating 5.04):**

Hi, my name is E. And this is my university story. For all my high school years, I felt I was 72% of myself in everything. In OSS, I won engineering with only 72% of my capacity. One of my biggest regrets was attending prep school. But if you have a successful dad and perform like a racing horse, all this becomes a dream. And there you go, I started university like another year of high school. First lateral transfer dream, after 2nd midterms double major dream, then after first finals double minor dream, finally after the first exams of second term just hoping to continue in my department, and after the second term struggling to stay away from summer school. Then come the friends, shisha, backgammon – and hello, summer school. In the third year we met engineering lessons. And we got intimate in my 4th,5th,6th and 7th years. What can I do, one can't warm to those lessons easily. I'm E, and today it has been 7 years, 3 months, 2 weeks and 5 days since my graduation, I have been an engineer for 2,666 days, but all these 2,666 days, I have missed my school. I miss METU; I miss those days.

**Example of a high-comprehensibility text (mean comprehensibility rating 8.52):**

I am at the tram station and pick up my little daughter, who has just been on an excursion with her class. She gets out last, with hanging head and tears in her eyes. I ask the teacher what happened. He has no idea and tells me that she was fun and bright all day long. I ask my daughter and she sobs something about a dog. Then her friend comes along and explains that our neighbor's dog has died. He had to be put to sleep for health reasons, and she just found out. I take my daughter in my arms and say: "The dog had to be put to sleep, that was certainly the best thing for him. But I don't understand why you're so sad. You have hardly ever seen the dog and perhaps patted it twice." She replies: "Yes, that's true, but I heard it every day. He used to bark and I knew he was there. Now he's gone and I miss his barking."
